# The effect of chronic obstructive pulmonary disease on quality of life in smoking electrical technicians

**DOI:** 10.1192/j.eurpsy.2025.994

**Published:** 2025-08-26

**Authors:** I. Sellami, A. Abbes, R. Gargouri, R. Khemakhem, A. Feki, W. Feki, N. Bahloul, S. Kammoun, M. L. Masmoudi, M. Hajjaji, H. Ayadi, K. Jmal Hammami

**Affiliations:** 1 Occupationnal medecine; 2 Pneumology; 3 Rheumatology, CHU Hedi Chaker, Sfax, Tunisia

## Abstract

**Introduction:**

Chronic obstructive pulmonary disease (COPD) is an under-diagnosed disease. Screening for this disease, assessing quality of life and combating smoking are key factors in the overall management of this pathology.

**Objectives:**

Our study aims to assess the effect of COPD on quality of life in smoking electrical technicians and their motivation to quit smoking.

**Methods:**

We conducted a descriptive, analytical, cross-sectional survey to assess the impact of COPD on quality of life among smoking electrical technicians and their motivation to quit smoking. A questionnaire was administered during a COPD screening day. Smoking cessation motivation was assessed using the Richmond questionnaire. COPD screening was carried out using COPD screening self-questionnaire from the French National Authority for Health. The COPD Assessment Test (CAT) was used to determine the presence and severity of respiratory symptoms.

**Results:**

Our population comprised 24 male smokers. Motivation to quit smoking was low in half of the participants. Eleven participants (45.8%) were identified as being “at risk” of developing COPD. Cough, sputum and chest tightness were more frequent in participants at risk of developing COPD than in those not at risk, with a significant difference. The mean CAT score in participants at risk of developing COPD was 12.2 ± 9.7. The impact on quality of life in participants at risk of developing COPD was low and moderate to high in 45.5% and 54.6% of participants respectively. Bivariate analysis showed that motivation to quit smoking was not correlated with either the COPD self-screening score or participants’ quality of life.

**Conclusions:**

COPD impairs the quality of life of workers at the electricity and gas company. Training sessions on breathing and anti-smoking actions in the electricity and gas company must be directed towards smoking employees by ensuring comprehensive management will serve to prevent the onset of COPD.

**Disclosure of Interest:**

None Declared

